# Can programme theory be used as a 'translational tool’ to optimise health service delivery in a national early years’ initiative in Scotland: a case study

**DOI:** 10.1186/1472-6963-13-425

**Published:** 2013-10-20

**Authors:** Jennifer Eaves, Wendy Gnich

**Affiliations:** 1 Department of Public Health, NHS Fife, Cameron Hospital, Cameron Bridge, Leven, Fife KY8 5RG, UK; 2 Community Oral Health Section, Faculty of Medicine, University of Glasgow, Glasgow Dental School, 378 Sauchiehall Street, Glasgow G2 3JZ, UK

**Keywords:** Process evaluation, Formative evaluation, Programme theory, Implementation fidelity, Programme improvement, Oral health, Scotland, Childsmile

## Abstract

**Background:**

Theory-based evaluation (TBE) approaches are heralded as supporting formative evaluation by facilitating increased use of evaluative findings to guide programme improvement. It is essential that learning from programme implementation is better used to improve delivery and to inform other initiatives, if interventions are to be as effective as they have the potential to be. Nonetheless, few studies describe formative feedback methods, or report direct instrumental use of findings resulting from TBE. This paper uses the case of Scotland’s, National Health Service, early years’, oral health improvement initiative (Childsmile) to describe the use of TBE as a framework for providing feedback on delivery to programme staff and to assess its impact on programmatic action.

**Methods:**

In-depth, semi-structured interviews and focus groups with key stakeholders explored perceived deviations between the Childsmile programme 'as delivered’ and its Programme Theory (PT). The data was thematically analysed using constant comparative methods. Findings were shared with key programme stakeholders and discussions around likely impact and necessary actions were facilitated by the authors. Documentary review and ongoing observations of programme meetings were undertaken to assess the extent to which learning was acted upon.

**Results:**

On the whole, the activities documented in Childsmile’s PT were implemented as intended. This paper purposefully focuses on those activities where variation in delivery was evident. Differences resulted from the stage of roll-out reached and the flexibility given to individual NHS boards to tailor local implementation. Some adaptations were thought to have diverged from the central features of Childsmile’s PT, to the extent that there was a risk to achieving outcomes. The methods employed prompted national service improvement action, and proposals for local action by individual NHS boards to address this.

**Conclusions:**

The TBE approach provided a platform, to direct attention to areas of risk within a national health initiative, and to agree which intervention components were 'core’ to its hypothesised success. The study demonstrates that PT can be used as a 'translational tool’ to facilitate instrumental use of evaluative findings to optimise implementation within a complex health improvement programme.

## Background

### Theory-based evaluation

'Programme theory’ (PT) describes the process through which an intervention is hypothesised to impact on outcomes and the conditions under which this occurs; that is, it sets out how an intervention is expected to bring about change in a particular context [1,2].

Theory-based evaluation (TBE) encourages stakeholders to focus on PT by examining activities used to effect change and their linkages to intended outcomes [1,3]. Involving programme stakeholders in explicating PT is considered important by advocates of TBE. Stakeholder involvement is viewed as a means of increasing shared understanding of the programme, assisting implementation through critical reflection, instilling a sense of ownership of the evaluation and of increasing confidence in the attribution of change to previously agreed actions [1,4,5].

The results of the PT development process are often set out in a 'logic model’ (LM) [1,2]. Logic models provide a visual and diagrammatic representation of a programme that depict a plausible and intended trajectory from inputs and activities to outputs and outcomes [6]. Using PT as a framework to build an evaluation strategy directs focus to mechanisms of change, assumptions and risks to achieving outcomes [1]. The approach goes beyond simply evaluating whether or not an intervention worked to explain *why* it led to observed outcomes [2]. Without delineating and testing PT or assessing implementation fidelity, it is impossible to distinguish between 'implementation failure’ and 'theory failure’: the enduring problem of the 'black box’ [2,7].

TBE was developed to address perceived inadequacies of existing methods-oriented evaluative approaches when applied to complex interventions [5,8]. Numerous definitions of complex interventions exist. Frequently agreed characteristics include: interventions comprised of multiple interacting components (or causal strands); implemented at multiple sites; involving multiple stakeholders at different organisational levels; relying on difficult behaviours by those delivering or receiving the intervention; targeting numerous outcomes (with emergent outcomes likely) and where tailoring to local context is permitted [9,10].

It is well recognised that the increasing complexity of social interventions poses fundamental challenges for evaluation not least because: the resources required to comprehensively evaluate such interventions are substantial, the mechanisms of effect are so variable and often cannot be fully articulated prior to implementation, and the influence of context (including variation in delivery between areas and overtime) must be considered in the assessment of effectiveness [9-11]. Context (including characteristics of settings, participants and service providers), by design often under-considered in experimental approaches to evaluation, is central to TBEs [1,4].

Furthermore, TBEs are reputed to have wide-ranging benefits for programme implementers and evaluators. These include: eliciting a shared vision of programme aims, providing a guide for implementation, ensuring realistic objectives, aiding development of useful performance indicators [2,4] and facilitating service improvement [4,12]. However, considerable challenges exist when developing and using PT in the evaluation of complex interventions, not least, the resources required [5], the ability to achieve stakeholder engagement and consensus [5], and the ability to usefully represent a complex reality in a simple model [10]. Several critics remain unconvinced that it is feasible or helpful [13,14]. This paper focuses on the contribution of TBE to service improvement.

### Use of evaluative learning

Learning from research and evaluation has not been 'translated’ into policy directives, programmatic action or practice to the degree anticipated [15,16]. In light of the myriad of factors known to impact on evaluation use, including stakeholders’ receptiveness to findings and perceived need for action [17,18], political contexts [3,17], perceived quality of evaluations [17,18], methods used to communicate findings [17], and timeliness of findings [17], this 'translational gap’ is perhaps unsurprising.

Reviewing evaluations of health improvement initiatives in Scotland, Beeston and Halliday [19] note that evaluation findings are often not widely reported or used to inform future policy or programme development. Bonner [3] highlighted examples of programmes evaluated to be effective that were terminated and others deemed unsuccessful which continued to be funded, and reported that 'Health Action Zones’ in England were rolled out with no published evidence from earlier implementation. It is essential that learning from programme implementation is better used to improve ongoing delivery and to inform other initiatives, if interventions are to be as effective, and cost-effective, as they can be.

### TBE as a formative tool

There is increasing consensus that evaluators should play a role not only in assessing programme effects (summative evaluation) but also in facilitating service improvements (formative evaluation) [15,20]. TBE is advocated to support evaluators in this endeavour by facilitating identification and communication of areas where existing PT is weak [2] or where risks to proposed mechanisms of change exist [21], for example through involving stakeholders in reviewing programmes’ underlying assumptions. Stakeholder involvement can increase use of evaluation findings [22,23].

While TBEs are hypothesised to facilitate service improvement, there are few case examples reporting direct instrumental use of findings. Much TBE literature is conceptual or theoretical, with fewer examples describing operationalisation of the approach [24]. While there has been some recent improvement in this position, examples describing TBE in practice often focus on the initial process of developing LMs and present the resultant PT, with little information given about the use of PT to assess impact or improve delivery. Advocates of TBE advise that developing PT should be an iterative process, not a single output [2,4].

Reflecting on TBEs of Scottish Health Demonstration Projects, the Scottish Executive [25] found that providing formative feedback and identifying conflicts in stakeholders’ goals and priorities was useful in the early stages of programme development. However, examples of formative use of PT throughout an evaluation are less common. Sullivan and Stewart (p196) [5] state that although formative evaluation has 'become more visible’ in the United Kingdom (UK) context and 'there are examples of collaborative action-oriented evaluative research’ that 'too little attention is paid to the role of evaluative method such as Theories of Change at the stage of programme design’.

A literature search, covering the last decade and searching CINAHL, Embase, Medline, PsychARTICLES, PsycINFO and SocINDEX (through the EBSCO interface) for the term TBE and/or commonly used variants did reveal several UK based evaluations, with formative aims [11,26,27]. For example, adoption of a TBE approach in the evaluation of Health Action Zones in England was described as being intended to enable the evaluation to contribute to the process of learning [26]. However, none of the UK papers explicitly described the process through which formative feedback was provided or the mechanisms through which evaluative learning subsequently shaped programme development.

The search identified a small number of studies that specifically reported using LMs for programme improvement; all were conducted in the United States of America with the exception of one Canadian study. Donaldson and Gooler’s [28] TBE of an employment and health improvement initiative aimed to facilitate programme improvement through regular feedback to stakeholders, although no specific information regarding what was uncovered by reviewing implementation, what was fed back to stakeholders, or whether there were programme improvements, was provided. For Sielbeck-Bowen’s [29] TBE of a teenage pregnancy and parenting programme, PT was developed with programme planners and staff. Implementation was then compared with the PT which revealed areas where delivery did not match the theory. However, the author did not discuss how findings were fed back and findings had not been used due to programme restructuring.

Three further studies provided examples of formative improvement resulting from TBE activity: Page et al. [30] in the context of encouraging school leavers to enter healthcare professions; Hawkins et al. [31] in a sexual violence prevention programme; and Bracht et al. [32] in a prophylactic programme for a newborn respiratory condition in Canada. All developed a LM, reviewed delivery against it, uncovered discrepancies between the models and actual delivery and reported that the review process resulted in modification of programme activities, with Page et al. [30] and Bracht et al. [32] identifying that the changes were likely to impact positively on outcomes. While these examples described formative comparison of PT against ongoing delivery, all involved small-scale programmes, and the methods used to review implementation and communicate findings to the programmes were not fully described.

Coryn et al. [24] call for case study examples describing the use of TBE and how findings from such evaluations are used. This paper addresses the paucity of such literature using the case of Scotland’s early years' oral health improvement programme, Childsmile. The aims are to explore the feasibility of using TBE as a framework for providing feedback on programme implementation to programme staff; to explore whether this process can facilitate the use of evaluative findings for ongoing programme improvement; and to add to existing literature, transparently describing the methods used to facilitate the formative process.

### Childsmile

Although the oral health of Scotland’s children is now improving, inequalities in oral health and access to care remain [33]. Funded by the Scottish government, Childsmile, is a comprehensive programme of dental public health and targeted clinical initiatives for children which aims to improve children’s oral health, reduce inequalities and improve access to dental services. In 2008, an 'integrated programme’ comprising four components was rolled out (to varying timescales) across all 14 geographic NHS boards in Scotland [33]. 'Childsmile Core’ offers oral healthcare packs and daily supervised toothbrushing to all children attending pre-school establishments (nurseries), and Primary 1 and 2 schoolchildren in disadvantaged areas. 'Childsmile Practice’ supports families identified by health visitors (HV) as likely to benefit from oral health advice. Dental health support workers (DHSWs), lay workers employed by NHS boards, provide tailored oral health advice and facilitate children’s attendance at a dental practice. Tailored advice and clinical prevention continues in the dental setting, provided by dentists or extended duty dental nurses (EDDNs). 'Childsmile Nursery’ and 'Childsmile School’ provide clinical prevention (fluoride varnish application), delivered by EDDNs in targeted establishments, and identify children with dental care needs, in order to facilitate dental attendance.

The programme is overseen by the 'Childsmile Executive’, a committee responsible for policy and decision-making, comprising: two directors, three programme managers and a representative from the evaluation team. Operationally, coordinators plan and implement the programme within each NHS board (with some boards having more than one coordinator to oversee different geographical areas or programme components). Each NHS board employs DHSWs and EDDNs to deliver the programme in partnership with dental practitioners, HVs and educational establishments.

Childsmile shares many of the features of complexity described within the evaluation literature: Childsmile involves multiple stakeholders across multiple centres and has multiple and interacting aims, components, targets, processes and potential outcomes; Childsmile draws upon theories of change from prior initiatives and comprises long chains of hypothesised activity between inputs and outcomes, which rely upon multiple behaviours performed by those delivering or receiving the intervention; and the social and healthcare context in which Childsmile is embedded and local tailoring within NHS boards, influence its delivery and likely effectiveness, as do ongoing learning and changing political drivers [9,10,34].

In an attempt to address this complexity, Childsmile is subject to a comprehensive TBE, led by a team based at the University of Glasgow and supported by three NHS-based regional research teams. This paper draws upon process evaluation (PE) activity undertaken as part of Childsmile’s wider TBE [35].

## Methods

In November 2009, Childsmile’s evaluation team and programme stakeholders developed LMs setting out the programme’s intended activities and outcomes. Childsmile’s logic models are available online [35]. Childsmile’s PE aims to explore the extent to which 'actual’ delivery differed from 'intended’ delivery as depicted in the LMs. To achieve this, the evaluation examines routine quantitative monitoring data alongside qualitative accounts of delivery. This paper focuses on qualitative data collected through in-depth, face-to-face interviews and focus groups with strategic-level stakeholders and operational staff. This primary fieldwork was supported and contextualised by documentary review and observation at programme meetings and events.

Participants were identified using a purposive sampling strategy; stakeholders likely to provide information relevant to the PE’s aims were included in fieldwork to provide a depth and richness of data. Fieldwork was carried out between August 2010 and June 2011. Interviews were held with the programme’s directors, all three programme managers and two other strategic stakeholders with input into decision-making. Twenty-two coordinators in post at the time of fieldwork, covering all 14 NHS boards, were interviewed. Focus groups were conducted with operational staff: 71 DHSWs from all 14 NHS boards; and 27 EDDNs, covering six NHS boards. EDDNs from the remaining boards were not included due to the early stage of programme implementation in these areas. Fieldwork and analysis reported here pertains to a single tranche of an ongoing PE.

Interviews and focus groups aimed to gather participants’ views on whether the activities set out in the LMs were taking place as intended, as well as to identify 'active ingredients’ perceived to contribute to programme outcomes and uncover any perceived risks to the achievement of outcomes. LMs were used as a discursive prompt. Participants were encouraged to describe programme implementation in their NHS board in detail and to consider barriers and facilitators to delivery. Topic guides are available on request.

All interviews and focus groups were recorded, transcribed and coded using NVivo 8. Written consent was obtained from all participants. Transcripts were analysed using the interpretative analytical technique of 'constant comparison’ [36]. Analyses focused on: the extent to which activities were implemented as intended and the likely impact of discrepancies; whether, if implemented as intended, activities were still perceived to be capable of bringing about desired change; as well as any perceived risks to the attainment of desired outcomes and unintended consequences. Some thematic categories related to the formative aims (listed above) and were coded to a priori 'aims driven’ nodes. Other themes grounded in the data were coded to 'emergent’ nodes. Discursive questioning and agreement, through team meetings, ensured the non-selectivity, validity and reliability of the findings.

Findings were presented to programme stakeholders in a written report. Following distribution, key stakeholders (including Childsmile Executive members, coordinators and others with input into local or national decision-making, including Consultants in Dental Public Health and Clinical Directors of Dental Services) were invited to a facilitated 'feedback event’ which aimed to consider the findings and resultant learning for service improvement. Thirty-five stakeholders attended.

At the feedback event, PE findings were verbally presented to all stakeholders. Participants were then split into four focus groups, and each asked to consider a key activity thought by PE respondents to have deviated in some way from PT. Questions focused on whether PE findings fitted with individual’s perceptions of delivery; whether, if deviations from the LM were apparent, they had the potential to impact on outcomes; and whether the issue needed further consideration or action. Focus group discussions were analysed as before and key findings distributed to all participants including Childsmile Executive members.

On considering the findings, the Childsmile Executive provided a written response to the feedback. This confirmed where they thought national action was already in place to address the issues raised and considered whether future national or local action was appropriate in relation to those issues not already addressed. Responsibilities and timescales were assigned to actions. To assess the impact of the formative feedback cycle described, as illustrated by programmatic action, the Childsmile Executive’s response was reviewed alongside knowledge gained from ongoing PE activity.

Verbatim quotes used for illustration were anonymised. Those participating in individual interviews were randomly assigned a number; operational staff who participated in focus groups, are denoted by a randomly assigned letter representing their NHS Board and their organisational role.

The University of Glasgow’s Medical Faculty Ethics committee approved the evaluation of Childsmile. NHS clinical governance approval was obtained.

## Results

As PT is not intended to remain static [2,4] and Childsmile is an evolving programme, which had not completed national roll-out at the time of data collection, differences between PT and stakeholder accounts were to be expected and were not necessarily a threat to the achievement of intended outcomes. Nonetheless, participants’ accounts conveyed that activities were being delivered “in the main” as planned. This suggests that the initial process of developing the PT in conjunction with stakeholders was successful in representing the intended programme of activity comprising 'Childsmile’.

However, considerable between-board variations in delivery were evident. Analyses suggested that differences had their origins in the stage of roll-out reached, and that the nationally developed 'blueprint’ for delivery (as depicted in Childsmile’s LMs) was not perceived as being intended to provide a rigid protocol for local delivery. Decisions shaping local implementation were reported to be largely at the discretion of individual NHS boards and strategic stakeholders viewed the freedom to adapt programme activities to each board’s unique context as an essential component of Childsmile’s PT.

In keeping with TBE and a formative approach, this paper focuses on four key activities (depicted in Childsmile’s LMs) which stakeholders described as *not* being delivered as intended: referral of newborns, provision of family support by DHSWs, follow-up of children not regularly attending dental services and nursery and school toothbrushing. Each of these activities was perceived (by stakeholders and the authors) to be critical elements of Childsmile’s PT, with the potential to influence outcomes. For each activity, a description of related PT is provided, followed by a brief synopsis of PE findings describing the extent to which participants perceived delivery of the activity to match PT. Conclusions and recommendations derived from discussions of PE findings at the feedback event and an assessment of the extent to which findings were used by the programme to optimise delivery are then presented for each activity.

### Referral of newborns

Childsmile’s PT states that HVs will routinely link all newborn babies to Childsmile through existing national child health systems. Families identified to have particular needs are directed to DHSWs for oral health advice and support to attend a dental practice. Others are directed straight to practice.

HV referrals were perceived as fundamental for engagement in the programme. However, participants reported variation in the extent to which HVs referred children to the programme. Some boards had well-established referral procedures, while others in earlier stages of roll-out were still in the process of establishing links with HVs. In some areas with no DHSWs, HVs referred children directly to dental practices. There were between-area differences in who was referred to DHSWs; in some areas, all newborns were referred, in others just those deemed to need additional support received a referral.

Several participants advocated HVs assessing families’ needs, to allow DHSWs to focus on supporting those most in need:

We can’t spread ourselves out too thinly, we need to concentrate on the needy ones.

(DHSW, Area T)

Mixed views were expressed about relationships between Childsmile and HVs. Some participants reported good links and received adequate numbers of referrals, while others highlighted difficulties in engaging HVs:

They don’t understand the programme, so they don’t refer in, it’s really hard to get them on board, and if they’ve got shortages within their team, the last thing they’re thinking about is referring to us.

(DHSW, Area S)

At the feedback event, participants emphasised the need to promote Childsmile and build relationships with HVs to increase awareness of local referral processes. Considering this feedback, the Childsmile Executive indicated that a board-level, as opposed to national response was appropriate, since successful referral depended on local-level partnerships between Childsmile staff and HVs. However, programme managers endeavoured to support boards in this task. The Childsmile Executive also noted that action was underway to include a check of a child’s dental registration status within the national Child Health Surveillance Programme assessment that takes place with all children aged 24–30 months in Scotland, serving to raise awareness about Childsmile among HVs. Priority was placed on this work.

### Provision of family support by DHSWs

Childsmile’s PT states that DHSWs will support targeted families in home or community settings. It is intended that DHSWs will contact referred families when their child is aged three months to provide oral health advice and assistance to register with a dentist. DHSWs may provide ongoing support if required.

In the majority of NHS boards, DHSWs supported (or intended to support) families via home visits. Elsewhere, it was planned that DHSWs would support families in other community settings due to reservations about the likelihood that vulnerable families would accept home visits, and concerns about staff-lone working. Both approaches fitted with the described activity in the LM.

However, another model involved directing all families to attend a dental practice when their child reached six months of age, with no support provided by DHSWs (although additional support would be provided if families failed to attend appointments). This model was used as an interim measure in boards where no DHSWs were in post. In these cases, a key component of Childsmile was missing; families might not receive oral health advice until their child was six months old, by which point they may have developed teeth and begun to be weaned.

Participants described engaging families as challenging. It was not always possible to contact families referred and some refused to participate despite repeated approaches.

Ensuring DHSWs were well-prepared to engage families and provide them with support was discussed. Some staff members (whose role was previously limited to supporting the Core and Nursery and School components) were thought to be apprehensive about undertaking home visits. One participant explained:

You’re taken out of your comfort zone to do a home visit.

(Dental Nurse, Area K)

Not all staff had opportunities to prepare for this new aspect of their role before commencing home visits:

I’ve not been on a home visit, I’ve not met my health visitors, I’ve not been introduced to them.

(DHSW, Area T)

Participants also identified variations in the amount of support given to families, such as the number of visits provided. Ongoing support via multiple visits was advocated for families with greater needs:

Some of them have got so many other issues before they even think about children’s teeth, it does take a few visits before you finally get through to them what you’re trying to say.

(DHSW, Area U)

HVs’ input on the level and type of support required by each family was thought to be important to tailor service provision. While DHSWs in one area visited families every six months, not all DHSWs provided ongoing support. Some participants reported that limited capacity had resulted in DHSWs being unable to support families to the degree necessary. In some areas, initial visits took place when children were aged six months or older. Participants viewed this as unsatisfactory, as dietary advice could not be provided before weaning.

During the feedback event, concerns were expressed that home-visiting was not provided in every NHS board. Several participants identified home-visiting as the preferred model, although others suggested that the content of contacts was more important than their location.

It was noted that some DHSWs’ discomfort with home-visiting had influenced local decisions about how support was provided to families. Participants suggested that additional training, to prepare DHSWs to support families, and impart further knowledge about eliciting behaviour change, was needed. Following the event, programme managers contacted coordinators to acknowledge concerns among staff about home-visiting, They highlighted the need for, and gave backing to, local approaches to ensure DHSWs were appropriately trained and supported. Since the event, several boards have implemented local training for DHSWs, focusing on communication and approaches to behaviour change.

### Following-up children not regularly attending dental services

'Surveillance’ was perceived by stakeholders as an important aspect of Childsmile’s PT. It is intended that children who do not regularly attend a dentist will be followed up. Dental practices are requested to inform a child’s DHSW if they fail to attend (FTA) two consecutive appointments, and parents of children identified in nursery or school as not registered with a dentist, or as requiring dental care, should be informed of their child’s needs.

Participants had mixed views about the extent to which this activity was delivered. Following-up children who FTA dental appointments was thought to work well in some cases; in others, there was uncertainty about whether practices referred all non-attending children back to DHSWs:

…[some practices have] *never ever got back to me to say that these people that you’ve referred in have not attended…they’re not getting back to you to say, so that you can go and chase them up.*

(DHSW, Area F)

It was also thought that dental practices might not inform DHSWs about families who attended their initial appointments but failed to attend subsequent appointments.

Once they received information about missed appointments, DHSWs in all areas aimed to contact families to offer further support. However, follow-up was reported to be time-consuming and limited DHSW capacity was reported to be restrictive:

…[we have] *a lot of fail to attends and I really don’t know how much time or how many visits the dental health support workers are actually going to follow those up, at the moment I would suspect they’re not doing many.*

(Respondent 5)

Capacity within dental services also affected follow-up:

We were so short of appointments that Childsmile children were being cancelled and re-scheduled, but nowhere to re-schedule them. So some, maybe, [they] have been lost.

(Dental Nurse, Area I)

Participants were also unclear whether those children who were not referred to a DHSW (but instead facilitated straight to practice) registered and continued to engage:

It’s up to the parent then to make their next dental check-up, which I’m quite concerned about because…we don’t know if the parents are all taking their kids back.

(Respondent 26)

Similarly, participants were uncertain whether parents who were notified that their child required dental care (identified through Nursery and School) attended a dentist. Childsmile staff were not informed of subsequent attendance; participants recalled examples of children who, although identified on several occasions as having potential dental issues, did not attend a dentist and whose dental health was perceived to be deteriorating:

It’s been a referral every time we’ve seen this kid… but I don’t feel like anything happens after that…

(Dental Nurse, Area U)

At the feedback event, participants acknowledged that Childsmile’s surveillance systems were “work in progress”. High FTA rates were highlighted by several participants as having the potential to impact on outcomes. It was noted that families failed to attend their appointments despite being issued reminders. However, participants recognised that actions to address FTAs would have to fit with dental practices’ own protocols. Participants questioned the extent to which dental practices would follow up persistent non-attenders (identified as being resource-intensive but yielding little income).

Participants emphasised the need to communicate to dental practices the importance of informing DHSWs of all children who missed two appointments. Gaps in communication between dental practices and DHSWs were thought to be evident. Although, following the event, the Childsmile Executive indicated that local action was appropriate, programme managers requested information about local protocols for dealing with FTAs from all areas and reviewed these to identify any gaps requiring further action. National information is now collected from NHS boards to ensure that protocols are in place for dental practices to communicate FTAs to DHSWs, as part of routine monitoring of boards’ delivery of oral health improvement activities linked to Scottish Government funding.

In recognition of NHS boards’ varying procedures for following-up children identified as requiring assessment and dental care through Nursery and School, the development of a national protocol was suggested. It was acknowledged that this would have to fit with local systems. At the time of review, production was being considered by the Childsmile Executive.

### Nursery and school toothbrushing

Childsmile’s PT states that there will be daily supervised toothbrushing in every nursery in Scotland and in Primary 1 and 2 classes in targeted primary schools (situated in disadvantaged areas). National Standards guiding the toothbrushing programme state that children will be supervised (by a member of school staff) while brushing daily. Local Childsmile staff supply toothbrushes and toothpaste, train school staff to supervise toothbrushing and monitor establishments’ delivery of the programme once per term.

Participants viewed the toothbrushing programme as key to contributing to intermediate and long-term outcomes:

Supervised toothbrushing involves teaching a lifetime skill, and encouraging good habits, to enable the child to think about the part they can play in taking care of their own oral health.

(Respondent 25)

However participants raised concerns that a potential unintended consequence of the toothbrushing programme was that parents might not brush children’s teeth at home:

My worry with the toothbrushing is that some of these families are going to think 'oh the kids are doing it at school we don’t need to bother at home’.

(DHSW, Area O)

The need to emphasise that participation in the toothbrushing programme did not negate the need for parents to establish twice-daily toothbrushing at home was underscored.

Participants also revealed that some targeted establishments declined to participate in the toothbrushing programme. Lack of engagement and negative attitudes among education staff were reported to be barriers to implementation:

I find in my schools, it’s them that’s got this barrier up…the class teachers, it’s them that’s sort of 'I haven’t time for this, we’re supposed to be teaching kids, we’re not supposed to clean their teeth’.

(DHSW, Area O)

Additionally, some participants were aware of establishments that had agreed to participate but had not initiated toothbrushing:

We know they’re not doing it, cause we’ll put brand new toothbrushes in at the beginning of term, and you go in to change them and they’re still all brand new.

(DHSW, Area E)

or alternatively were not toothbrushing every day:

We have nursery schools who, yeah, they brush when you’re there, but they might not brush the other four days of the week.

(Respondent 15)

As day-to-day delivery of toothbrushing is undertaken by school staff, it is not possible for Childsmile staff to check delivery every day.

The finding that targeted establishments had declined to participate was discussed at the feedback event. Participants identified that local Childsmile staff should establish links with education departments and individual establishments to encourage participation, although it was thought to be preferable for establishments to participate willingly rather than being coerced. Discussions re-emphasised the need for well-engaged education staff, with ongoing, tailored support required for all establishments. Reductions in classroom assistant posts due to budgetary constraints were also thought to have had a negative impact, with some teaching staff unwilling to take on supervision of toothbrushing. A means of monitoring school activity was regarded as important.

Following the feedback event, Childsmile’s coordinators have continued to attempt to engage non-participating establishments at a local level. No direct national action has been taken to address this since the consensus was for action by individual boards. However, concerns around the unintended consequences of brushing in the nursery and school setting have been taken up nationally; issues with implications for stakeholder communication, identified through the PE, have been considered in the development of the programme’s national communications strategy.

The need for improved monitoring of the frequency and quality of delivery was also agreed through the discussions. In response to this feedback, the Childsmile Executive has reviewed how the toothbrushing programme is monitored and put a national audit in place.

## Discussion

Looking inside the 'black box’ of interventions is important to assess whether they are implemented as intended [2,4]. Implementation fidelity cannot be assumed when assessing effectiveness as inadequate implementation may explain unachieved outcomes [37]. By comparing Childsmile’s PT with 'delivery-in-reality’, the approach described affords essential interpretation to the programme’s summative evaluation [7]. However, this paper focuses upon the formative utility of the TBE approach, exploring whether iteratively reviewing Childsmile’s PT facilitates use of PE findings for service improvement.

While activity detailed in Childsmile’s LM was largely perceived to be implemented as intended, the TBE approach facilitated a strategic focus on activities which were not implemented as intended, or which could be improved. The approach served to highlight areas where 'differences’ might pose risks to the achievement of outcomes. Such 'areas for action’ largely originated from between-board variations in delivery. Interventions implemented in the 'real world’ often require adaptation; indeed interventions that do not undergo tailoring to their local context may be a poor fit to their setting, limiting their success [7,15]. In Scotland, local adaptation of initiatives is encouraged by government funders [38]. Damschroder et al. [15] explain that interventions can be conceptualised as having 'core components’ (essential, indispensable elements of their PT) and an 'adaptable periphery’ (adaptable elements, structures and systems related to the intervention and organisation into which it is being implemented) which may be tailored without adversely affecting outcomes. It is essential to an intervention’s success that those responsible for delivery agree which components of PT are 'core’ and which are 'peripheral’, in order that tailoring to context (such as Childsmile’s tailoring to individual NHS board settings) retains key 'active ingredients’ [7,15].

Within Childsmile, some local adaptations were perceived to have deviated from the 'core features’ of PT to the extent that they impacted on the programme’s ability to effect change; for example, families in some boards were not offered any tailored support by DHSWs irrespective of identified needs. The TBE approach provided a platform, first for emphasising the need to clarify which components of the intervention, or features of those components, were 'core’, and secondly for discussing and agreeing these essential characteristics.

A key contribution of this paper lies in the methodologically robust assessment of the 'instrumental use’ of PE findings, in the main absent from prior literature. TBE provided a comprehensive framework to explore fidelity to proponents’ vision of Childsmile, and for reporting potential risks and unintended consequences back to stakeholders. The outcomes-focussed approach afforded a platform for discussions, facilitated shared understanding and language, and gave immediate relevance to process findings, all facilitative of stakeholder engagement and use. Inherently iterative, the approach was able to capture ongoing programme development.

The TBE approach described clearly supported service improvement. There were numerous examples of national action taken by the programme as a result of formative feedback. However, not all formative recommendations were acted upon. Some issues, highlighted as a potential risk to achievement of outcomes, were not immediately amenable to programmatic action; for example, lack of DHSWs to provide family support resulting from local boards’ recruitment policies was deemed to be outside of the programme’s control. Action to address this issue was therefore not thought possible. The methods employed did, however, heighten awareness of the issue.

In other cases it was thought that national action was inappropriate, and that local responses to the issues raised were required. That local actions to address identified issues were suggested fits with the general finding that differences in implementation resulted from decision-making and tailoring at a local level, and that solutions needed to consider local systems. For example, between-board variations in the extent to which HVs referred newborns to Childsmile were thought to be best addressed by local action to further develop partnership-working between HVs and Childsmile staff in individual boards. While TBE highlighted the need for action, it did not in itself resolve differences in opinion as to the extent to which national direction was required as opposed to local action. While some stakeholders perceived a board-level response as sufficient, others cautioned that this had to be directed by clear national guidance and, where appropriate, supportive national actions. For complex, multi-setting initiatives like Childsmile to be effective, it is essential that responses to issues raised by TBE achieve the right balance between national direction and local adaptation.

A number of caveats should be borne in mind when interpreting findings. First, this paper assessed the strategic, national response to PE feedback; it is beyond the scope of reported work to elucidate the extent to which staff within local NHS boards took tailored action in response. Since operational staff took part in facilitated discussion of PE findings (at the feedback event), received recommendations following the feedback event, and were provided copies of strategic stakeholders’ responses to the issues raised, and since local action was often favoured, there is likely merit in exploring this further.

Second, this paper focussed on direct 'instrumental use’ of PE findings [18,39]. We did not systematically investigate other types of research and evaluation use described in the literature. It is plausible that the findings influenced knowledge and understanding about the programme without direct action (conceptual use) [18,40]; that findings were used to persuade or justify existing positions (symbolic use) [18,39,40], or that stakeholders’ behaviour and thinking was changed as a result of learning obtained through participating in the evaluation (process use) [18,39,40].

Third, the PE involved programme decision-makers and operational staff; it did not include any service users (children or care-givers). Proponents of TBE suggest that those targeted by an intervention should be included or consulted during the process of ongoing PT development and testing [2,5]. While, to date, research staff capacity has precluded service user consultation as part of Childsmile’s PE, considering families’ views may lead to the identification of otherwise unexplored risks associated with Childsmile’s PT which could be addressed to improve programme delivery (for example user accessibility and acceptability). Future PE activity will test Childsmile’s PT through consultation with service users. It will also examine the extent to which boards used the PE findings to make improvements to local delivery and further explore alternative types of use.

Fourth, we have analysed and fed back subjective perceptions about successes and barriers to implementation; longer-term impact evaluation will afford an objective assessment of effectiveness. Producing evaluative evidence of this type requires a lengthy duration and it is widely recognised that programmes require feedback within shorter timeframes than is often possible through the 'typical’ research cycle [41,42]. The two-year cycle of formative research reported here was in itself viewed as protracted by stakeholders eager for feedback, necessitating the need for interim cycles of 'key issues’ being fed back, based, due to the time constraints, on preliminary analysis. Nonetheless, the methods described demonstrate the feasibility of a feedback cycle designed to fulfil a national programme’s need for early formative information. The approach fits alongside approaches such as utilization-focused evaluation, which advocate that evaluations should be responsive to programme stakeholders’ needs [22]. Impact evaluation findings will be shared in due course.

Fifth, in terms of the generalisability of findings, it is possible that Childsmile stakeholders’ willingness to engage in formative processes and their eagerness to contribute to, and use, an evidence-base may not translate to all sectors or geographical contexts. The transparency with which methods are reported in this paper, affords the opportunity for others to replicate our methodology and test the generalisability of our approach to other initiatives and contexts. Differences between the American and UK context have been reported to impact on how TBE can be used, particularly the extent to which all stakeholders can be engaged in theory development and testing [5]. This paper provides a much needed example outside of the American context, demonstrating that despite challenges suggested within the evaluation literature, a national TBE, implemented in Scotland, with appropriate resources, can achieve sufficient stakeholder consensus to develop an adequate representation of programme complexity, and maintain a level of ongoing stakeholder engagement, facilitative of programme improvement

Finally, these findings represent a snapshot at one point in time, when NHS boards were in the process of rolling out Childsmile. It would be unrealistic and unwelcome for a complex national service initiative such as Childsmile not to develop and improve over time, thus the picture of delivery may now be different. While feedback was observed to affect action, the case-study design does not allow direct attribution of change to the formative process.

## Conclusions

This paper addresses recent calls for case study examples describing the use of TBE approaches. It adds to current literature by describing the methods used to explore utilisation of formative evaluation feedback within a TBE in the Scottish context, and by using robust qualitative methodology to explore the extent to which feedback resulted in action to improve delivery. In contrast to the majority of published studies, it focuses on the iterative process, which, after the initial development of PT, should be part of any TBE. The results suggest that direct instrumental use of PE findings can be facilitated by using PT as an evaluative framework. It is concluded that PT can be used as a 'translational tool’ to facilitate service optimisation in a complex, national, NHS health improvement programme. With tailoring to the local context, the methodological approach documented is likely to be of use to those responsible for planning, implementing and evaluating complex public health initiatives internationally.

## Abbreviations

TBE: Theory-based evaluation; PT: Programme theory; NHS: National health service; HV(s): Health visitor(s); DHSW(s): Dental health support worker(s); EDDN(s): Extended duty dental nurse(s); PE: Process evaluation; LM(s): Logic model(s); UK: United Kingdom; FTA: Failure to attend.

## Competing interests

The authors declare that they have no competing interests.

## Authors’ contributions

WG conceived of the study. JE undertook fieldwork. Both JE and WG contributed to study design, thematic analysis, drafting and revising the manuscript and have approved the final version.

## Authors’ information

WG is a research fellow in Dental Public Health and evaluation at the Community Oral Health Section, University of Glasgow. JE is a researcher employed by NHS Fife. Both authors are members of a larger evaluation team funded to undertake a comprehensive evaluation of Childsmile.

## Pre-publication history

The pre-publication history for this paper can be accessed here:

http://www.biomedcentral.com/1472-6963/13/425/prepub
